# A Simulation Analysis of Nanofluidic Ion Current Rectification Using a Metal-Dielectric Janus Nanopore Driven by Induced-Charge Electrokinetic Phenomena

**DOI:** 10.3390/mi11060542

**Published:** 2020-05-27

**Authors:** Weiyu Liu, Yongjun Sun, Hui Yan, Yukun Ren, Chunlei Song, Qisheng Wu

**Affiliations:** 1 School of Electronics and Control Engineering, Chang’an University, Middle-Section of Nan’er Huan Road, Xi’an 710064, China; liuweiyu@chd.edu.cn (W.L.); qshwu@chd.edu.cn (Q.W.); 2 School of Mechatronics Engineering, Harbin Institute of Technology, West Da-zhi Street 92, Harbin 150001, China; rykhit@hit.edu.cn (Y.R.); sclei@hit.edu.cn (C.S.); 3 State Key Laboratory of Robotics and System, Harbin Institute of Technology, West Da-Zhi Street 92, Harbin 150001, China

**Keywords:** induced-charge electrokinetic phenomena, ion diode, concentration polarization, metal-dielectric Janus ion-exchange medium, nanofluidics

## Abstract

We propose herein a unique mechanism of generating tunable surface charges in a metal-dielectric Janus nanopore for the development of nanofluidic ion diode, wherein an uncharged metallic nanochannel is in serial connection with a dielectric nanopore of fixed surface charge. In response to an external electric field supplied by two probes located on both sides of the asymmetric Janus nanopore, the metallic portion of the nanochannel is electrochemically polarized, so that a critical junction is formed between regions with an enriched concentration of positive and negative ions in the bulk electrolyte adjacent to the conducting wall. The combined action of the field-induced bipolar induced double layer and the native unipolar double layer full of cations within the negatively-charged dielectric nanopore leads to a voltage-controllable heterogenous volumetric charge distribution. The electrochemical transport of field-induced counterions along the nanopore length direction creates an internal zone of ion enrichment/depletion, and thereby enhancement/suppression of the resulting electric current inside the Janus nanopore for reverse working status of the nanofluidic ion diode. A mathematical model based upon continuum mechanics is established to study the feasibility of the Janus nanochannel in causing sufficient ion current rectification, and we find that only a good matching between pore diameter and Debye length is able to result in a reliable rectifying functionality for practical applications. This rectification effect is reminiscent of the typical bipolar membrane, but much more flexible on account of the nature of a voltage-based control due to induced-charge electrokinetic polarization of the conducting end, which may hold promise for osmotic energy conversion wherein an electric current appears due to a difference in salt concentration. Our theoretical demonstration of a composite metal-dielectric ion-selective medium provides useful guidelines for construction of flexible on-chip platforms utilizing induced-charge electrokinetic phenomena for a high degree of freedom ion current control.

## 1. Introduction

With the rapid development of nanoscience and nanotechnology, interfacial mechanical phenomena have received unprecedentedly increasing attention from the microfluidic society, due to their favorable scaling with greater surface-to-volume ratios in small-scale systems [1,2]. Among them, diffuse-charge dynamics inside a Debye screening layer represents an important polarization effect at the contact interface between a solid surface and neighboring electrolyte solution [3,4,5]. During this electrokinetic process, a native free surface charge density is firstly attached to the channel walls, and then a thin electric double layer (EDL) occupied by mobile counterions has to be induced to make the combined surface-charge/double-layer system electroneutral [6]. Within the thin EDL, the concentration of counterions dominates that of coions. Since a diffuse screening cloud appears as a consequence of equilibrium between electrophoretic delivery and Brownian diffusion of ionic charge carriers near charged solid surfaces, the thickness of the EDL depends sensitively on the background ion number density, and can range from less than 1 nm for concentrated solutions to beyond 100 nm in the dilute electrolyte [7,8].

Inside a nanometer-size space (at least one dimension reaches the nanometer scale), the Debye screening layer stands for an important part of the entire volume, and thereby the inhomogeneous surface conductivity within the thin boundary layer overwhelms in front of bulk conductivity [9,10]. For instance, in the presence of an appreciable extension of the EDL, the surface charge density natively adsorbed on channel walls results in a high degree of symmetry breaking in the ion contents, with a global concentration difference between cations and anions. In this way, only counterions are allowed to pass through nanoscale channels, while the axial motion of coions is forbidden. That is, the electrokinetics for nanofluidic channels with characteristic size commensurate with the thickness of EDL is governed by surface charge, and behaves in a completely different manner from electrokinetic phenomena in microfluidic devices, since double-layers overlap inside nanofluidics [11,12,13]. Most importantly, because of the ion-selective delivery relative to the channel wall charge of opposite sign, electric current flux along the length direction is highly controllable by surface conduction within nanofluidic channels, while it is almost impossible to achieve this in microscale ducts where the electrophoretic transport of ionic species is dictated by bulk Ohmic conductance [14,15,16].

Considering its advantages in ion current control, up to now, a variety of nonlinear on-chip ion circuit devices have realized the functionalities of ion current diodes [17,18,19], transistors [19,20,21], magnifiers [22], and biosensing [23,24,25] by adjusting ion number densities inside nanofluidic space. As for the field of ion diodes, rectification implies the electric current for applied voltage of one direction is much higher than that for voltage in the opposite direction. Ion current rectification demands the presence of a net surface charge density on nanochannel sidewalls, which can be conventionally achieved either by chemical attachment of charged units [26,27,28,29] or in a field-effect-tunable multiple electrode system by imposing gate voltages to the gate electrodes embedded on the outer surface of the nanochannel [30,31,32]. In the former, the most effective rectification is realizable by a bipolar membrane that incorporates a critical junction between regions with surface charges of opposite polarity [33,34,35,36,37,38,39,40,41,42,43]. Such systems, termed “ionic diodes”, are usually characterized by static charge magnitude due to chemical processes, so it is difficult to alter their ion-selective property once the nanofabrication procedure is finished. In the latter, though we can achieve a dynamic adjustment on the local zeta potential of the nanofluidic diode by field-effect control, the introduction of gate electrodes complicates the device architecture and adds a certain degree of difficulty to the fabrication process [44].

**Figure 1:**
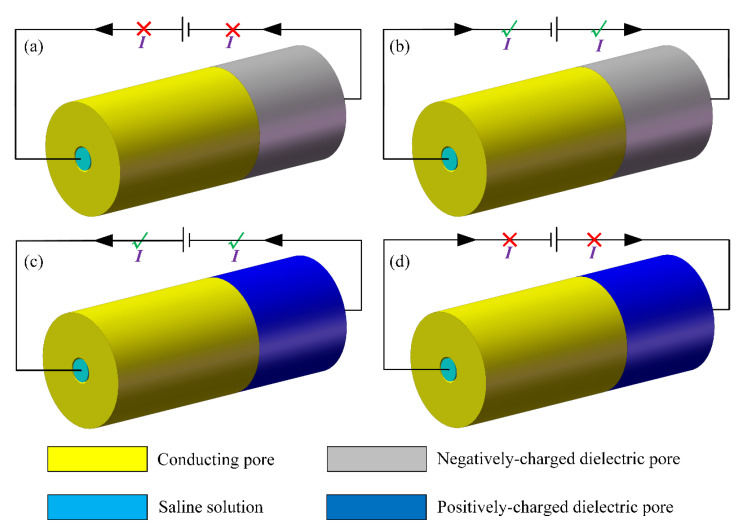
A 3D sketch of the metal-dielectric Janus nanopore, which serves as an active nanofluidic ion diode on the basis of ICEK-enabled internal concentration polarization, under an external DC voltage supply of reverse polarity. (**a**,**b**) The rectification trait of the asymmetric Janus nanopore with a cation-selective dielectric nanopore on the right side, (**a**) as the external DC electric field passes through the nanopore from the conducting side to the dielectric side, the ionic device works in the “off” state, and the forward electric current is forbidden, (**b**) with a reversal in the voltage polarity, the ion circuit platform transits to the “on” status, in which the backward current is well switched on. (**c**,**d**) Rectification characteristic of the nanoscale ion diode with an anion-selective dielectric nanopore on the right end, (**c**) for a positive DC bias, the nanoscale ion diode operates in the on state, in which the forward current flux is enhanced to great extent by the ion-enrichment zone formed at the midchannel, (**d**) for a negative DC bias, the functional ion platform transits to the off status, in which the backward electric current is switched off due to the ion-depletion zone induced at the critical junction between the enriched concentration of cations and anions.

To overcome the drawbacks of the two kinds of popular mechanisms for ion current rectification, we report herein a unique category of rectifying principle by a delicate combination of the two existing techniques. The proposed nanopore diode has a simple two-electrode configuration, which features surface charge profiles that are induced by an externally-applied electric field via electrochemical polarization of the inner surface of a conducting metallic nanochannel. The density of the induced surface charge on the conducting end is linearly proportional to the voltage difference along the nanopore direction according to the theory of induced-charge electrokinetics (ICEK) [45,46,47,48,49,50,51,52,53], and once in combination with a natively-charged dielectric nanopore gives rise to a desired ion-current rectification behavior (Figure 1a–d). Reversal of the rectification polarity occurs once the dielectric nanopore with a negative wall charge (Figure 1a,b) is replaced by one with a positive wall charge (Figure 1c,d). Due to a mirror symmetry of the two rectification situations, we focus on the case with a negatively-charged dielectric end (Figure 1a,b), while paying less attention to its counterpart (Figure 1c,d). Due to the unique rectification mechanism of the metal-dielectric Janus nanopore developed herein, it may be further extended for osmotic energy conversion application as well, in which an electrical current is produced by a horizontal salinity gradient even in the absence of external voltage excitation, as has been recently reported by the Yeh group using a Janus membrane [54,55]. Although some nanoscale ion diodes using induced-charge electroosmosis (ICEO) have been developed, it is rare to make use of the combined effect of ICEK and permanent interfacial polarization to construct an ionic circuit element, in which the missing of any one mechanism will disable the diode functionality [56]. It is firmly believed that ion current rectification driven by ICEK at the nanometer scale is going to inspire interdisciplinary research in soft matter, analytical chemistry, and osmotic energy harvesting in the near future.

## 2. Methods

### 2.1. Basic Theory of Bipolar Floating Electrode for Rectification with a Janus Nanopore

**Figure 2:**
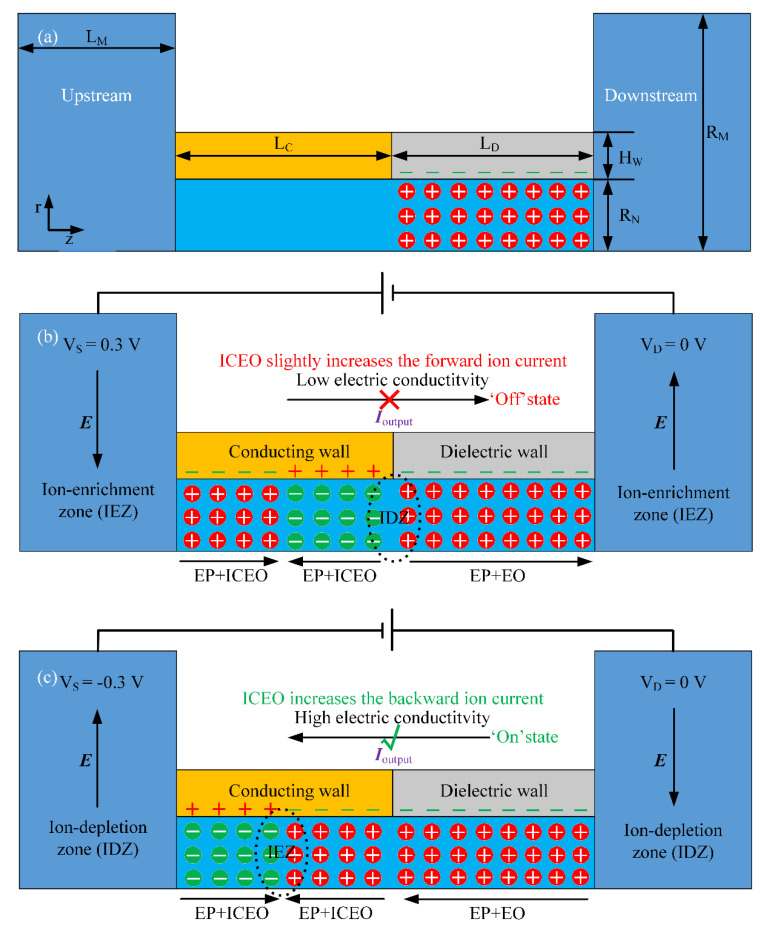
A 2D cross-sectional schematic diagram of the nanofluidic ion diode exploiting induced-charge electrokinetic (ICEK)-mediated internal ion concentration polarization (ICP) in a metal-dielectric Janus nanopore for achieving reliable ion current rectification in the r-z axisymmetric coordinate system, in which the dielectric wall is negatively charged and serves as a cation-selective medium. (**a**) Geometry and size of the nanofluidic device, as well as the spatial distribution of counterions and surface charge inside the nanopore without external voltage excitation. (**b**,**c**) In the presence of an external DC electric field, the combination of a bipolar induced double layer (IDL) within the metallic nanochannel and unipolar cations within the negatively-charged dielectric nanochannel gives rise to a voltage-adjustable non-uniform space free charge distribution, whose electrochemical transport along the nanopore results in (**b**) an ion-depletion zone (IDZ) in the midchannel for a positive DC bias and thereby the “off” working state of the nanofluidic ion diode with the forward current being greatly weakened, or (**c**) an ion-enrichment zone (IEZ) in the center of the conducting wall for a negative DC bias and therefore the “on” working state of the ion circuit platform with the backward electric current being greatly enhanced.

**Figure 3:**
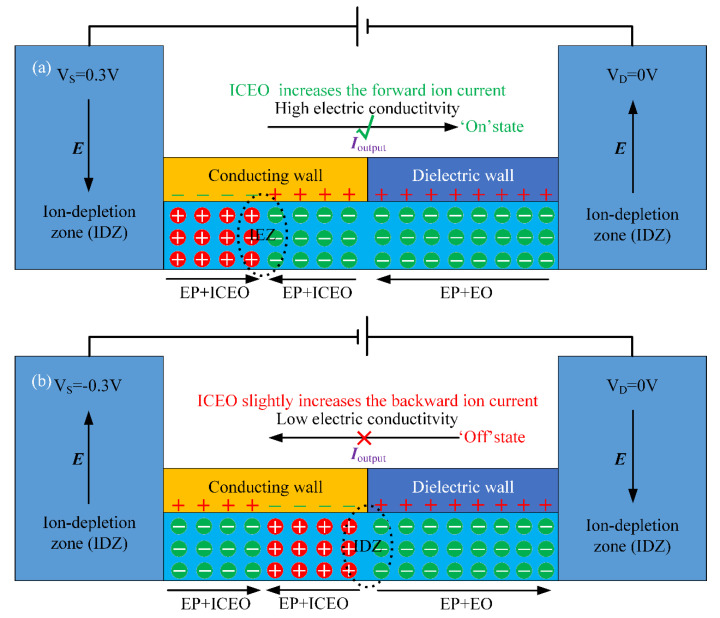
A 2D cross-sectional schematic diagram of the nanofluidic ion diode exploiting ICEK-mediated internal ICP in a metal-dielectric Janus nanopore for achieving reliable ion current rectification in the r-z axisymmetric coordinate system, in which the dielectric wall is positively charged and serves as an anion-selective medium. Under an external DC electric field, the combination of a bipolar induced double layer (IDL) within the metallic nanochannel and unipolar cations within the positively-charged dielectric nanochannel gives rise to a voltage-adjustable non-uniform space free charge distribution, whose electrochemical transport along the nanopore results in (**a**) an ion-enrichment zone (IEZ) in the center of the conducting wall for a positive DC bias and therefore the “on” working state of the ion circuit platform with the forward electric current being greatly enhanced and (**b**) an ion-depletion zone (IDZ) in the midchannel for a negative DC bias and thereby the “off” working status of the nanofluidic ion diode with the backward current being greatly weakened.

When a voltage difference is imposed on the two electrode terminals on both ends of the metal-dielectric Janus nanopore, charge carriers within the metallic nanochannel redistribute to cancel out the local electric field. According to charge conservation, the induced charge on the inner surface of the conducting pore has a bipolar pattern in essence, with the anodic end including excess negative charge carriers, and the cathodic end including surplus positive charge carriers (Figure 2b,c and Figure 3a,b). Once placed in a saline solution, the ideally polarizable conducting surface modulates local charge concentrations by diffuse charge dynamics, in terms of the formation of sequential regions with reinforced anion and cation number densities. 

The capability for floating metal conductors to induce a bipolar induced double layer (IDL) has been well supported by experimental observation of a pair of counter-rotating electroosmotic vortexes on top of an unbiased metal strip under an external DC/AC electric field [47,48,57,58,59]. Field-induced Debye screening of the floating electrode is also utilized in electrochemical science as a bipolar electrode for generating Faradaic ion concentration polarization for enrichment of charged molecules [60,61,62,63,64]. In the present study, we demonstrate by direct numerical simulation that electrochemical polarization of a conducting nanochannel located at the entrance of a metal-dielectric Janus nanopore permits voltage-biased control of local charge concentrations for the development of a nanofluidic ion diode.

### 2.2. Chip Geometry and Device Operation Principle

Basic geometric configuration of our nanofluidic ion diode is a metal-dielectric Janus nanopore (Figure 1) of 2*R_N_* in inner diameter, 2(*R_N_* + *H_W_*) in outer diameter, and *L_N_* = *L_C_* + *L_D_* in length, which bridges two microfluidic chambers of *R_M_* in diameter and *L_M_* in length on both sides (Figure 2a). The cylindrical Janus nanochannel is composed of two sequential parts, including a metallic nanopore and natively-charged dielectric nanopore (Figure 1) of an identical height of *H_W_*, and is stuffed with an electrolyte solution. The conducting section of the Janus nanopore has a non-negligible length of *L_C_*, as compared to the length *L_D_* of the dielectric component, so as to achieve a voltage-based control over the specific ion contents along the thin channel. In this way, under an imposed DC electric field from the source (S) to drain (D) terminals, any translating motion of ionic charge carriers occurs permanently through the cylindrical nanochannel surrounded by a Janus wall. As a consequence, the flow path across S-D terminals casts the role of the output fluidic channel for the nanofluidic system. The usage of a metal-dielectric Janus wall makes ion conductivity at the critical junction between zones of enriched cations and anions (induced by diffuse charge dynamics of an ideally polarizable conducting surface) govern the ultimate electric current flowing across the composite nanopore.

In the current analysis, the inner wall of the metallic channel is hypothetically uncharged in the absence of any external voltage supply. On the contrary, the dielectric channel is supposed to have a uniform distribution of static surface free charge of a negative (Figure 2) or positive polarity (Figure 3) from permanent interfacial polarization, leading to a native EDL filled with cations (Figure 2) or anions (Figure 3) within the dielectric nanopore. Since the two situations with oppositely-charged counterions inside the dielectric channel can all achieve ion current rectification, albeit with a reversal of the electric current direction (Figure 2c and Figure 3a), we focus mainly on the device operation principle utilizing a negatively-charged metal-dielectric Janus nanopore, as shown in Figure 2. 

Besides the native EDL full of cations within the dielectric nanopore, the conducting nanochannel mounted at the pore entrance can be readily polarized by a DC electric field imposed along the channel length direction, which gives rise to additional induced counterions of a bipolar nature under the covering region of the metal pore via the physical principle of induced-charge electrokinetics (ICEK) (Figure 2b,c) [65,66,67,68,69,70,71,72,73]. Namely, more cations than anions are induced on the anodic side of the conducting wall, while excessive anions are induced on the cathodic side of the conducting channel, regardless of the specific polarity of the applied DC bias (Figure 2b,c). Consequently, a junction between sequential regions of enriched cations (anions) and anions (cations) is induced around the contact border between the metallic and dielectric nanochannels by the applied DC voltage. The functionality of such a field-induced ion junction resembles that of the fixed charge junction in a classical bipolar membrane for current rectification [74]. Even so, when considering the field-induced nature of the bipolar IDL that serves as an important component of the rectification junction, our approach possesses the merit of voltage-based control, making the metal-dielectric Janus nanopore much more flexible than the traditional bipolar membrane for developing a high-performance nanofluidic ion diode. 

Taking the device with a negatively-charged dielectric end for example, as shown in Figure 2, the D terminal within the right reservoir is always grounded. For a positive DC bias, e.g., *Vs* = 0.3 V, imposed at the S terminal immersed in the left electrolytic port, the rightward DC electric field lines throughout the Janus nanopore induces electrophoretic transport of field-induced anions from the cathodic to the anodic side of the conducting channel, and that of native cations from the dielectric channel to the cathodic microchamber, due to a mismatching counterion polarity at these virtual contact interfaces (vertical dotted lines in Figure 2b). This gives rise to an ion-depletion zone (IDZ) in the midchannel, which lowers the ion flux toward the downstream electrolytic port to great extent, so that the forward electric current is shut down under a positive *Vs*, namely, the "off" working state (Figure 2b). In the meantime, a convection current due to charge motion driven by ICEO has a tendency to enhance slightly the forward electric current, which exerts an adverse influence on the device rectification performance.

On the contrary, with a negative DC bias, e.g., *V_S_* = −0.3 V, lower than the zero voltage of the D terminal, the applied electric field reverses in polarity and points from the right to left microchamber (Figure 2c). This causes the induced negative ions at the cathodic conducting channel, as well as the positive ions within the negatively-charged dielectric channel, to move into the field-induced bipolar junction in the center of the conducting wall. This effectively gives rise to an ion-enrichment zone (IEZ) at the ICEK-based bipolar junction. In this way, the solution conductivity toward the S terminal is elevated greatly, and the backward Ohmic current can be well developed under a negative *V_S_*, i.e., the “on” working state (Figure 2c). At the same time, ICEO streaming inside the confined nanopore tries to enhance the backward ion current flux, which improves the rectification coefficient. At the same time, although there is a large number of ions in the IEZ adjacent to the ICEK-enabled bipolar junction, both cations and anions become concurrently depleted in the electrolytic ports on both sides for mass conservation (Figure 2c). By adjusting buffer ion strengths inside the nanoscale duct, the ICEK-based nanofluidic ion diode using a metal-dielectric Janus nanopore provides new opportunities for advancing the field of on-chip integrated circuit platforms.

### 2.3. Hypothesis and Approximation Applied in the Present Study

According to the operation principle of nanopore ion diode, two distinct kinds of electroconvection, including traditional linear electroosmosis (EO) and nonlinear ICEO, arising from the Coulomb force within the native EDL, and the field-induced bipolar Debye layer, respectively, coexist in the functional nanofluidic device. Considering the complexity of the synthetic flow field from combined EO and ICEO, we have to quantify the diode performance by numerical simulation. 

For the convenience of mathematical analysis, the KCl aqueous electrolyte of 1:1 binary symmetric monovalent ionic species is chosen as the working fluid of reference in the present study. Though the nanochannel is of a finite size in simulation, our nanoscale ion diode is highly scalable, so long as an elongation of the fluid path is accompanied by a greater length of the metal-dielectric Janus wall. The volume of the microchambers on both sides could be freely extended as well according to real demand, provided that a larger DC bias was applied throughout the system to render the nanoscale ion diode operate efficiently at a larger dimension. Although our numerical computation focuses on ICEK-mediated ion transport for a standard KCl saline solution, a small inner radius of the nanopore readily furnishes a strongly overlapped EDL for a wide range of buffer conductivities. Consequently, the nanofluidic ion diode of asymmetric Janus walls can be operated at a high rectification performance even for polyelectrolytes.

### 2.4. Mathematical Description

On the basis of continuum mechanics, ion motion within water is governed by a strong coupling model of Poisson–Nernst–Navier–Stokes equations. For the sake of simplicity, the physical description for a binary symmetric electrolyte of 1:1 monovalent ion species is carried out a priori such as KCL saline solution. Local ion concentration in the working fluid is related to the in-situ electrical potential by the Gauss law in a conductive medium:

(1) $$\rho_{\mathrm{free}}=\nabla \cdot \left(\varepsilon_{f}E_{f}\right)=-\varepsilon_{f}\nabla^{2}\phi_{f}=F\left(c_{+}-c_{-}\right)$$

where *F* represents the Faraday constant, and $\varepsilon_{f}=80\varepsilon_{0}$ the permittivity of water, and $\varepsilon_{0}$ that of air. From preceding equation, the net free charge density $\rho_{\mathrm{free}}$ is determined by the concentration difference between cations c_+_ and anions c_−_.

On the contrary, it is supposed that there is no space free charge within the Janus wall, and as a consequence, electrostatic potentials within the conducting wall ($\phi_{C}$) and dielectric wall ($\phi_{D}$) are both controlled by the Laplace equation:

(2) $$\nabla^{2}\phi_{C}=0$$

(3) $$\nabla^{2}\phi_{D}=0$$

Ion mass conservation meets the law given by the Nernst–Planck equation:

(4) $$\frac{\partial c_{+}}{\partial t}+\nabla \cdot J_{+}=0\;\mathrm{with}\;J_{+}=-\mu_{+}Fc_{+}\nabla \phi_{f}-D_{+}\nabla c_{+}+uc_{+}$$

(5) $$\frac{\partial c_{-}}{\partial t}+\nabla \cdot J_{-}=0\;\mathrm{with}\;J_{-}=\mu_{-}Fc_{-}\nabla \phi_{f}-D_{+}\nabla c_{-}+uc_{-}$$

where *D*_+_ and *D*_−_ denote the diffusivity of ionic charge carriers, respectively, with $\mu_{+}$ and $\mu_{-}$ being the electrophoretic mobility for corresponding ion species. According to the Einstein relation, *D*_+_/*μ*_+_ = *D*_−_/*μ*_−_= k_B_*TF*/*q*, in which k_B_ stands for the Boltzmann constant, *T* the ambient temperature, and *q* the elementary charge.

Taking away Equation (5) from Equation (4), the transient charge conservation equation can be obtained:

(6) $$\frac{\partial \rho_{\mathrm{free}}}{\partial t}+\nabla \cdot \left(\sigma_{f}E_{f}-D\nabla \rho_{\mathrm{free}}+u\rho_{\mathrm{free}}\right)=0$$

where $\sigma_{f}{=F}^{2}\left(\mu_{+}c_{+}+\mu_{-}c_{-}\right)$ defines the local electric conductivity of the leaky dielectric electrolyte. With the knowledge of the Gauss law $\rho_{\mathrm{free}}=\nabla \cdot (\varepsilon_{f}E_{f})$, the condition of continuity of electric current density is explicitly given by:

(7) $$\nabla \cdot J=0\;\mathrm{with}\;J=\sigma_{f}E_{f}+\varepsilon_{f}\frac{\partial E_{f}}{\partial t}-D\nabla \rho_{\mathrm{free}}+u\rho_{\mathrm{free}}$$

where ***J*** denotes the total current flux, and is comprised of four different current components, including Ohm conduction due to electromigration of charged ions, displacement current due to dielectric polarization, diffusion current in a gradient of ion concentration, and convection current due to charge transport in viscous flows, as expressed by the four sequential terms in Equation (7). In the present analysis, since a time-invariant DC voltage offset is applied across the nanopore length direction, the displacement current which is proportional to the time derivative of the external electric field is negligibly small compared to the conduction counterpart. Accordingly, the conduction current flux is a conservative vector field because of the objective condition of ∇⋅J=0.

Fluid motion in the nanopore ion diode is determined by the modified Navier–Stokes equations including a source term of electrostatic body force:

(8) $$\rho \frac{\partial u}{\partial t}+\rho \left(u\cdot \nabla u\right)=-\nabla p+\eta \nabla^{2}u+\rho_{free}E_{f}$$

(9) ∇⋅u=0

where *p* stands for the hydraulic pressure, ***u*** the flow velocity field, ρ the liquid mass density, and η the dynamic viscosity of water. The additional term $\rho_{free}E_{f}$ is the space Coulomb force density acting on the free charge within both the native and induced EDL, which are well resolved by the conjugating conditions imposed at the dielectric wall/liquid interface and metallic wall/liquid interface. In the meantime, the Maxwell stress exerted on any electrical phase interface is not incorporated in the multiphysics numerical model, in that all the solid entities in the nanopore ion device are physically fastened on an insulating substrate.

Since the electroosmotic pump flow rate along the ion-selective nanochannel is usually much larger than traditional electroosmosis in microchannels dictated by bulk conductivity, it is reasonable for us to disregard Debye screening on insulating charged walls of microchambers on both sides [75,76]. In spite of this, the extended space charge layer (ESCL) induced in IDZ can be still captured by our numerical simulation, since the physical origin is in regard to non-uniform surface conduction of excessive counterionic charges within the nanopore, rather than charging of microchannel walls, as has been explicitly considered in the mathematical treatment. The overlooking of diffuse charge dynamics in microfluidic reservoirs prevents excessive fine meshes from appearing near the chamber walls on both sides. In this way, the numerical modeling can be readily conducted even in the presence of a confined computer resource. 

### 2.5. Boundary and Conjugating Conditions

Since the nanopore has a cylindrical shape with excellent space geometric symmetry, we establish herein a 2D axisymmetric computational model for the proposed nanoscale ion diode with ICEK-mediated current control. Then, direct numerical simulations are carried out to understand the mechanical behavior of the ICP interface developed within the confined nanochannel, under the simultaneous polarization of the metallic walls and charged dielectric walls due to the DC bias applied in the nanopore axial direction. Because macroscopic electrokinetic equations are still adaptable for typical channel sizes no less than 1 nm, it is suitable for us to employ the classical theory of continuum mechanics to describe ion transport behavior inside a metal-dielectric Janus nanopore. We take into account the convective delivery of ion species under the coaction of 1st EO (conventional linear DC electroosmosis), and nonlinear induced-charge electroosmotic (ICEO) streaming. The 1st EO arises from the fixed surface charge chemically adsorbed on the nanopore dielectric walls, and the Coulomb force acting on the natural Debye screening charge within the original EDL. On the other hand, ICEO appears as a consequence of electrochemical polarization of the conducting wall driven by the external DC voltage, and the resulting electrostatic body force within the field-induced diffuse screening cloud [46,77,78,79,80,81,82]. Both kinds of electroconvective fluid motion are well embodied by correct boundary conditions as well as the body force term in Equation (8). Considering the strongly coupled nature of the static electric field (Equations (1)–(3)), ion transport (Equations (4) and (5)), and electrohydrodynamic flow (Equations (8) and (9)), it is an important task to deal with this boundary-value issue.

#### 2.5.1. Boundary and Conjugating Conditions for the Electric Field

To obtain the electric field in the fluid, metallic wall, and charged dielectric wall, Equations (1)–(3) have to be calculated simultaneously, with proper conjugating conditions at the inner interfaces and boundary conditions on the outer surfaces of the ion diode device. Since the net conduction current decays to zero normal to nanopore dielectric walls from the two adjacent phases on both sides, static-free charge density can be supposed to be chemically adsorbed at the surface of a dielectric solid entity immersed in the electrolyte, which lays the theoretical foundation of diffuse-charge dynamics for insulating charged surfaces. So, an arbitrary distribution of surface free charge density $\sigma_{\mathrm{free}}$ is convincingly prescribed at the inner wall of the dielectric nanopore in direct contact with the buffer medium:

(10) $$\sigma_{\mathrm{free}}=-\varepsilon_{f}\frac{\partial \phi_{f}}{\partial n}+\varepsilon_{D}\frac{\partial \phi_{D}}{\partial n}$$

At the same time, the electrostatic potential has to be continuous across the phase interface:

(11) $$\phi_{f}=\phi_{D}$$

In stark contrast with the fixed charge polarization on dielectric walls, ICEK occurs at the ideally polarizable surface of metallic nanopore, and is described by the continuity of displacement current across the phase interface between the conducting wall and electrolyte solution:

(12) $$\varepsilon_{f}\frac{\partial \phi_{f}}{\partial n}=\varepsilon_{C}\frac{\partial \phi_{C}}{\partial n}$$

where $\varepsilon_{C}$ is the real permittivity of the metallic wall. The phenomenon of chemical adsorption of Cl^−^ ions addressed in [83] was not considered in the present study. Even so, this effect can be incorporated into the current simulation model by adding a negative interfacial charge density at the metal/electrolyte interface in the conjugating condition of Equation (12). By doing so, another uniform component of cations will appear within the metallic nanopore, and tend to destroy the field-induced bipolar charge pattern within the EDL by making the polarity of counterions more uniform along the nanochannel length direction (for example, the anions are replaced by cations in Figure 2b,c). As a result, the phenomenon of ion adsorption on the metallic walls may lower the rectification performance of the nanofluidic ion diode, and is not considered in the current analysis to avoid such a negative influence.

At the source and drain electrode terminals, the electrical driving voltage is determined by an external function generator:

(13) $$\phi_{f}=V_{S}\;\mathrm{at}\;\mathrm{the}\;S\;\mathrm{terminal}$$

(14) $$\phi_{f}=0\;\mathrm{at}\;\mathrm{the}\;\mathrm{drain}\;\mathrm{terminal}$$

If complete Debye screening takes place on the surface of the source and drain electrode terminals, almost all of the applied DC voltage will drop across the EDLs at the electrode/electrolyte interface, leaving no electric field in the bulk fluid any longer [47,56,84]. However, the appearance of an exchange current density from phase transfer of electrons may deteriorate the structure of EDLs formed on the electrode surface, and therefore permit electric field leakage outside of the EDL due to incomplete capacitive charging. So, it is suitable to apply the Dirichlet condition at both electrode terminals.

The normal component of the total electric current flux vanishes on the insulating walls of microchambers due to complete Debye screening right outside the native double-layer:

(15) $$n\cdot \nabla \phi_{f}=0$$

#### 2.5.2. Boundary Conditions for Ion Transport 

As for calculating the space distribution of salt concentrations (Equations (4) and (5)), since the ionic charge carriers cannot penetrate from the buffer medium into adjacent solid phases, the normal flux of both cations and anions disappears on nanopore walls:

(16) $$\left(-\mu_{+}Fc_{+}\nabla \phi_{f}-D_{+}\nabla c_{+}\right)\cdot n=0$$

(17) $$\left(\mu_{-}Fc_{-}\nabla \phi_{f}-D_{-}\nabla c_{-}\right)\cdot n=0$$

The combined Equations (16) and (17) indicates that the total conduction current is completely inhibited by the two-phase contact interface:

(18) (σE−D∇ρ)⋅n=0

Invoking the Gauss law, a scaling analysis of the current insulation condition Equation (18) implies the Debye screening charges exist chiefly within a characteristic length scale of $\lambda_{D}=\sqrt{D\varepsilon /\sigma }$ away from the electrical solid/liquid interface.

The ion concentration of c_+_ = c_−_ = c_0_ is prescribed at the S and D terminals, which is consistent with actual conditions. Electrophoretic mobility *μ*_+_ = *μ*_−_ = 8.2 × 10^−13^ s·mol·kg^−1^ is linearly related to the diffusion coefficient of ion species *D*_+_ = *D*_−_ = 2 × 10^−9^ m^2^·s^−1^ via Einstein relation inside the domain of KCl aqueous electrolytes.

#### 2.5.3. Boundary Conditions for Electroosmotic Flow Field

The flow dynamics of electroosmosis is regulated by conservation laws in Equations (8) and (9). We set the S and D terminals on both ends of the fluidic system as open boundaries. No slip-wall condition is imposed on the walls of the two microchambers as well as the central Janus nanopore.

#### 2.5.4. Numerical Simulation

Electrochemical ion transport in the metal-dielectric Janus nanochannel bridging the microchambers on both ends is rather complicated. It is necessary to establish a numerical model for providing an in-depth understanding of a series of electrokinetic phenomena resulting in ion current rectification along the asymmetric nanochannel. The unidirectional propagating behavior of the diffusion boundary layer (DBL) in an H-shaped dual-channel microfluidic ion diode has been theoretically studied in previous literature [44,85,86], which enables a thorough comprehension on the active interplay among ICP and electroosmotic flow for electroconvective extension of DBL. However, the physical process encountered herein appears to be more intricate. The ideally polarizable metallic nanopore introduces the phenomena of ICEK, namely, an induced double layer (IDL) of bipolar counterions appears within the conducting nanochannel on application of the DC voltage offset. The synergy of the dipolar IDL within the metallic channel and unipolar EDL within the negatively-charged dielectric channel makes the counterions unevenly distributed inside the nanopore (Figure 2b,c). This lays the fundamental physic mechanism upon which internal ICP due to nanoscale ICEK and the resultant diode functionality are based.

In order to describe correctly the ICEK-based effects, numerical calculations applying microscopic conservation laws routinely require an extremely fine grid distribution, with the maximum mesh size no more than one-thirtieth of the Debye screening length within the metal-dielectric Janus nanopore (please refer to the Figure S1 in the Supplementary Information for the mesh-independence test of the simulation results). For such, researchers usually simulate fluidic chambers at micrometer dimensions, which is quite beneficial in terms of keeping the calculations within the limit set by available computer resources [14,87,88,89,90,91,92]. A background ion concentration of c_0_ = 0.1 mM is utilized for both positive and negative ions at the early time, leading to an EDL thickness of 30.5 nm, and matches the inner diameter *H* = 30 nm of the Janus nanopore. In this way, both the unipolar EDL and bipolar IDL can well stretch and run throughout the nanopore lateral dimension for achieving effective ion current rectification. Moreover, extra-fine triangular meshes of 0.05 nm in edge size were utilized at the entrance and exit of the nanochannel interfacing microchambers on both sides, for resolving the existence of an in-situ extended space charge layer (ESCL) wherein electroconvective ion transport plays an important role. When the device is exposed to an external DC electric field, an ion enrichment or depletion zone appears at the critical bipolar-charge junction adjacent to the midchannel. So, the Debye length will decrease or increase in pace with the variation of local ion number density, which is adverse or beneficial for obtaining a higher computational accuracy.

### 2.6. Scaling Analysis

We then conduct a preliminary scaling analysis for getting an approximate condition for the rectification behavior to occur. Separated by the critical junction between the conducting and dielectric nanopore, the electrochemical transport mechanism within the two neighboring regions is completely different from one another. Under the Debye–Huckel limit and shallow channel approximation (*R_N_* ≤ λ_D_), the native fixed zeta potential (FZP) across the Debye screening cloud due to fixed surface charges on the walls of dielectric nanopore is given by:

(19) $$\zeta_{\mathrm{fixed}}=\frac{\sigma_{\mathrm{free}}R_{N}}{\varepsilon_{f}}$$

On the other hand, an induced zeta potential (IZP) of a bipolar charge pattern due to ICEK of the metallic walls of the Janus nanopore appears as a consequence of the horizontal DC electric field, whose characteristic magnitude rather than the specific value can be approximately expressed by Equation (20) in the presence of complete electrochemical polarization and shallow channel approximation (*R_N_* ≤ $\lambda_{D}$):

(20) $$\zeta_{\mathrm{induced}}=\frac{E\cdot L_{C}}{2}\frac{1}{1+\frac{\varepsilon_{f}H_{W}}{\varepsilon_{C}R_{N}}}$$

Because the electrical impedance of the nanochannel is orders of magnitude larger than that of the two microchambers, almost all of the imposed DC voltage offset drops across the thin duct, so we have a good estimation of the background electric field strength:

(21) $$E=\frac{V_{0}}{L_{C}+L_{D}}$$

Substituting Equation (21) into Equation (20), a relationship between the bipolar induced zeta potential and applied DC bias is obtained:

(22) $$\zeta_{\mathrm{induced}}=\frac{1}{2}\frac{V_{0}}{1+L_{D}/L_{C}}\frac{1}{1+\varepsilon_{f}H_{W}/\varepsilon_{C}R_{N}}$$

To realize strong enough internal ICP zone, it is necessary to generate a non-uniform distribution of bipolar counterions at the critical junction in the midchannel in Figure 2b. So, the negative ionic charges induced by the cathodic side of the conducting wall should be greater than a threshold defined by the unipolar cations inside the negatively-charged dielectric wall:

(23) $$\zeta_{\mathrm{induced}}\geq \zeta_{\mathrm{fixed}}$$

Combining Equations (19), (22), and (23), an analytical judgment criterion for sufficient ICP to occur for achieving reliable ion current rectification is formulated as below:

(24) $$\frac{1}{2}\frac{V_{0}}{1+\alpha }\frac{1}{1+\varepsilon_{f}H_{W}/\varepsilon_{C}R_{N}}\geq \frac{\sigma_{\mathrm{free}}R_{N}}{\varepsilon_{f}}$$

where $\alpha =L_{D}/L_{C}$ is a nondimensional parameter that characterizes the length of the metallic channel relative to that of the dielectric channel. Equation (24) can be further reduced to a simpler form for an ideally polarizable conducting channel with $\varepsilon_{C}\gg \varepsilon_{f}$:

(25) $$\frac{V_{0}}{2\left(1+\alpha \right)}\geq \frac{\sigma_{\mathrm{free}}R_{N}}{\varepsilon_{f}}$$

An idealized working state may be realizable when the left-hand side (LHS) exceeds the right-hand side (RHS) of Equation (24) in terms of an unbalance between the induced zeta potential (IZP) and fixed zeta potential (FZP). In practice, however, it is difficult to find a precise combination of various parameters to satisfy Equation (24). So, conducting simulation analysis prior to experiments serves as a good method of choice for getting an optimum rectification performance.

Though there is not an electrolyte conductivity dependence in Equation (24), another implicit condition for producing a sufficiently high Dukhin number is that the thickness of the diffuse screening cloud has to be commensurate with the nanopore inner diameter:

(26) $$\lambda_{D}=\sqrt{\frac{{D\varepsilon }_{f}}{{2\mu F}^{2}c_{0}}}\to 2R_{N}$$

Accordingly, the original ion concentration of the background saline solution should approach a critical value:

(27) $$c_{0}\to \frac{{D\varepsilon }_{f}}{{8\mu F}^{2}R_{N}^{2}}$$

Invoking a typical nanopore radius of *R_N_* = 15 nm, we acquire the condition that c_0_ should approach 0.1 mM as far as possible in addition to Equation (24), so that the Debye length matches the nanochannel thickness. In this sense, so long as the nanoscale ion device meets the conditions of Equations (24) and (27) simultaneously, the present ion circuit platform can operate normally as an efficient on-chip nanofluidic ion diode.

### 2.7. Quantification of the Rectification Performance

The device performance of the nanofluidic ion diode is characterized by the rectification factor γ, which is defined as:

(28) $$\gamma \left(V_{S}\right)=\left|\frac{I\left({-V}_{S}\right)}{I\left(V_{S}\right)}\right|=\frac{\iint_{\mathrm{outlet}}J\left({-V}_{S}\right)\mathrm{ds}}{\iint_{\mathrm{outlet}}J\left(V_{S}\right)\mathrm{ds}}\times 100\%$$

where *Vs* and −*Vs* within the parenthesis denote the value of corresponding variables for source voltage of opposite polarity, *I* the total electric current at the exit of the ion-selective nanopore, and *J* the ionic current density as given by Equation (7). For example, the rectification factor $\gamma \left(V_{S}=0.3V\right)$ at a given source voltage of *V_S_* = 0.3 V is equal to the specific ratio of I (*Vs* = −0.3 V) to I (*Vs* = 0.3 V).

## 3. Results and Discussion

### 3.1. Effect of Length Ratio of the Metallic Wall Relative to the Overall Nanopore Axial Extension

First and foremost, since the nanofluidic ion diode is operated on the basis of ICEK behavior of a metal-dielectric Janus nanopore, we should pay attention to the importance of the length proportion of the metallic wall compared to the overall axial extension of the asymmetric ion-selective medium. In this way, we are able to judge whether it is possible to produce a more reliable internal ICP interface in the midchannel by reconfiguring the ideally polarizable metallic end of the Janus nanopore. In the preliminary stage, an appropriate set of diverse geometrical and physiochemical parameters are arbitrarily given by: the length of the conducting nanopore *L_C_* = 600 nm, the length of negatively-charged dielectric nanopore *L_D_* = 400 μm, the total nanopore length *L_N_* = *L_C_* + *L_D_* = 1 μm, the thickness of nanopore wall *H_W_* = 100 nm, the inner radius of nanopore *R_N_* = 15 nm, the radius of the microchambers *R_M_* = 1 μm, the length of the microchambers *L_M_* = 1 μm, the source voltage *V_S_* = ±0.3 V, native surface charge density on the dielectric wall σ_free_ = −0.001 C/m^2^, and background salt concentration c_0_ = 0.1 mM, leading to a Debye length of the electrical double layer of λ_D_ = 30 nm, which can be right extended across the nanopore lateral direction with λ_D_ = 2*R_N_*.

**Figure 4:**
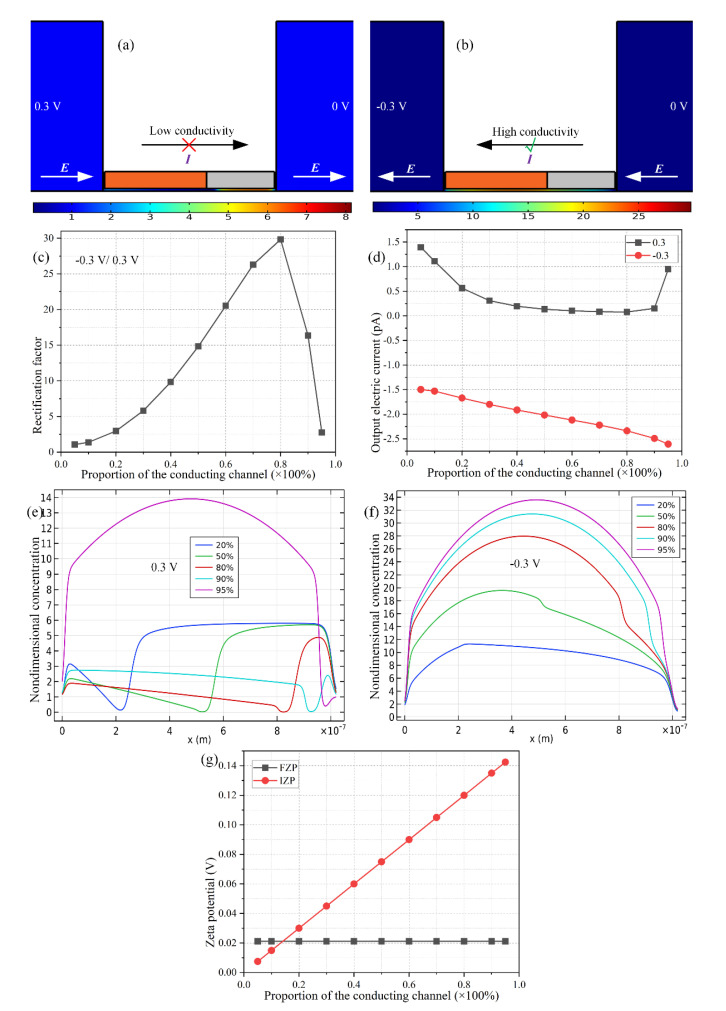
Influence of the length of the metallic nanopore with respect to that of the nanopore on the resulting rectification performance of the asymmetric metal-dielectric Janus nanopore, under a given parametric space of c_0_ = 0.1 mM, *Rn* = 15 nm, *V_S_* = ± 0.3 V, and *σ_free_* = −0.001 C/m^2^. (**a**) A surface plot of the nondimensional electrolyte concentration for the “off” working state, in which the forward electric current diminishes due to the IDZ formed at the bipolar junction between the metallic and dielectric wall. (**b**) A surface plot of the nondimensional electrolyte concentration for the “on” working state, in which the backward electric current is magnified due to the IEZ formed at another bipolar junction at the central portion of the metallic nanopore. (**c**) Rectification factor and (**d**) the forward and backward output electric current as a function of the length proportion of the metallic channel. (**e**) Ion concentration distribution along the centerline of the Janus nanopore for a positive DC bias, and (**f**) that for a negative DC bias. (**g**) A comparison of fixed zeta potential (FZP) within the charged dielectric nanochannel and induced zeta potential (IZP) within the metallic nanochannel as a function of the length proportion of the conducting nanopore.

The resulting salt concentration distribution under the coaction of the induced polarization of the metallic wall and native Debye screening of the negatively-charged dielectric wall in a horizontal static electric field provided by the DC bias across the S-D terminal pair is visually displayed in Figure 4a,b. Under a positive voltage *V_S_* = 0.3 V imposed on the source terminal, with the drain terminal being constantly grounded, as expected from the cross-sectional schematics in Figure 2b, a background electric field ***E*** towards the downstream outlet port produces an ion-depletion zone (IDZ) at the physical junction between the metallic wall and dielectric wall (Figure 4a), due to the simultaneous outward delivery of field-induced anions around the cathodic side of the metallic wall, and the natural cations within the dielectric nanochannel. The extremely low conductivity of the IDZ inhibits any output electric current ***I***_output_ toward the downstream D terminal, namely, the forward "off" operation state of the nanoscale ion diode (Figure 4a).

On the other hand, once the imposed voltage reverses in polarity, e.g., *Vs* = −0.3 V, an ion-enrichment zone (IEZ) is created at the field-induced bipolar junction in the middle of metallic nanopore (Figure 4b), which is consistent with the theoretical prediction shown in Figure 2c. This type of enrichment in ionic species reduces the electric impedance of the fluidic system and thereby greatly enhances the output electric current I_output_ towards the upstream S terminal, namely, the backward “on” working state of the nanofluidic ion diode.

The rectification performance of ionic current flux for the metal-dielectric Janus ion-selective medium is quantitatively calculated by direct numerical simulation, as a function of the specific length proportion of the ideally polarizable conducting channel. As shown in Figure 4d, the electric current flux is severely suppressed in the forward direction, while being greatly enhanced in the backward direction, which is in good agreement with the surface plot of salt concentration in Figure 4a,b. Importantly, there is an observable dependence of the ultimate rectification factor on the length of the conducting wall relative to that of the entire nanopore (Figure 4c). The rectification performance has a single peak point when the length proportion of the conducting nanopore β = 1/(1+α) reaches 80%. Any deviation of metallic wall’s length from this critical value of *L_C_* = 800 nm for a total nanopore length of *L_C_* + *L_D_* = 1 μm will decrease the device performance in ion current rectification. 

The above rectification trend as a function of the length of metallic wall (Figure 4c) can be well understood by the electric current shown in Figure 4d. With an increase of the length proportion of metallic wall from 0% to 100%, the backward current increases gradually (the red line in Figure 4d) due to a higher ion concentration in the IEZ at the middle of the metallic nanopore (Figure 4f), the location of which shifts from upstream to downstream at a larger value of the length of the metallic wall (Figure 4f). This enhanced degree of ICP in the regime of IEZ is due to an increase of the induced zeta potential (IZP) compared to the constant fixed zeta potential (FZP) for a longer metallic wall, as shown in Figure 4g, which increases mathematically with *L_C_* due to a larger polarization area of the ideally polarizable conducting surface, as given by Equation (22). 

Nevertheless, the forward ionic current is determined not only by the value of IZP, but also by the specific area of IDZ which almost diminishes for a relatively long metallic wall. As displayed in Figure 4d, as the conducting wall increases in length, the forward electric current first decreases due to a lower ion concentration in the IDZ at the bipolar junction between the metallic wall and dielectric wall from an enhanced value of IZP (Figure 4e,g). Nevertheless, once the metallic nanopore occupies 80% of the entire metal-dielectric Janus nanopore, the forward current no longer reduces, and instead, rises sharply even for a slight increase of the metallic wall length. As shown in Figure 4e, the IDZ cannot be healthily developed once the Janus nanopore tends to become a thoroughly conducting nanopore, due to a confined area around the Janus junction for IDZ to occur (the purple line in Figure 4e). Consequently, the forward current can only be well inhibited for an intermediate length of the metallic wall of 1/1 + α = 80%, when taking into account a trade-off between the degree of ion depletion and effective area for concentration polarization.

To this end, since a high rectification performance appears as a consequence of a large backward current and small forward current, it is necessary for us to construct an asymmetric Janus nanopore with an intermediate length proportion of the metallic wall (80%) for achieving the most efficient ion current rectification, in which both bipolar IDL due to ICEK and unipolar EDL due to fixed-charge polarization make important contributions to the diode functionality of the nanofluidic device. It is noteworthy that the optimal value portion of the metallic section may vary and deviate away from 80% once the fixed zeta potential or nanopore length makes a change. So, in practice, it is appropriate to adopt a neutral ratio (40%–60%), if it is required to achieve a moderate rectification performance even without conducting a numerical prediction in advance.

### 3.2. Effect of the Native Surface Charge Density of the Dielectric Wall

According to preceding simulation analysis, the bipolar IDL due to ICEK of the metallic wall and unipolar EDL of the dielectric wall act synergistically to induce the internal ICP interface, which serves as the main working principle of the present ion diode. So, an appropriate length proportion of the metallic wall *L_C_* = 600 nm (60%) is chosen from now on for further simulation investigations. In this way, a good compromise is realized between the degree to which ICP occurs and the area around the Janus junction available for the depletion of ionic species in the “off” working mode. 

**Figure 5:**
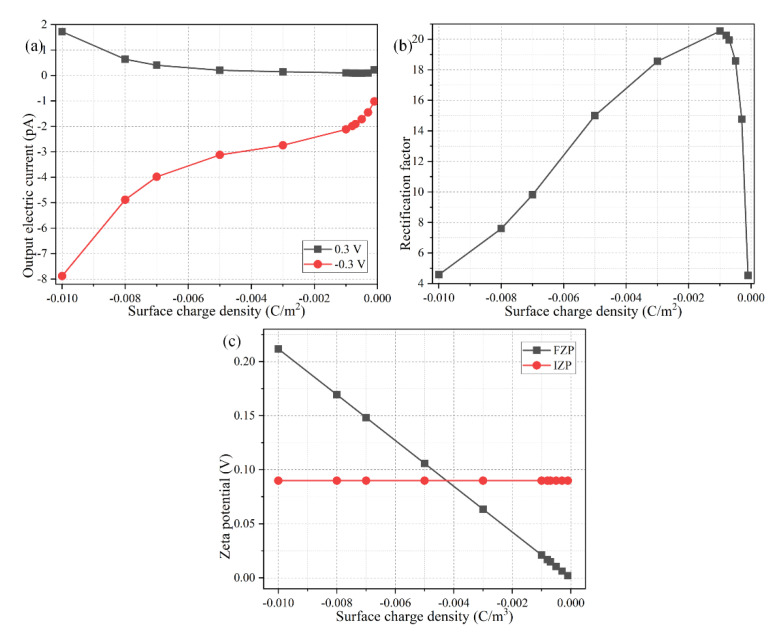
Effect of the native surface charge density of a negatively-charged dielectric wall on the device rectification performance, under a given parametric space of *R_N_* = 15 nm, *L_C_* = 600 nm, *V_S_* = 0.3 V, and c_0_ = 0.1 mM. (**a**) The forward and backward output electric current, and (**b**) the rectification factor, and (**c**) approximation of the zeta potential magnitude, as a function of the surface charge density.

Spontaneous Debye screening from the native surface charge on the dielectric wall generates a homogenous distribution of counterions, which are cations for a negative surface charge, as shown in the schematics in Figure 2a. From Equation (22), the variation of surface charge density σ_free_ has no impact on the value of IZP, which is consistent with the analytical approximation shown in Figure 5c. On the contrary, the value of FZP decays with a decreasing magnitude of the negative surface charge density, as predicted by the linear relation shown in Equation (19). As a result, the polarization degree due to ICEK increases with respect to the native Debye screening effect, as the surface charge density approaches zero from a negative value (Figure 5c). This implies an enhancement in the induced polarization of the metallic walls, leading to the generation of a more effective internal ICP interface adjacent to the bipolar Janus junction (forward “off” state, Figure 2b) or the center of the conducting wall (backward “on” status, Figure 2c) in the Janus nanopore. For such, the diode functionality of the nanofluidic device becomes optimal for a quite small value of the negative interfacial charge density (σ_free-ideal_ = −0.001 C/m^2^), as exhibited in Figure 5b. However, as the interfacial charge further decreases in magnitude and drops below the ideal value, the rectification factor starts to become negligibly small. That is, there is an ideal charging state of the dielectric wall of σ_free-ideal_ = −0.001 C/m^2^ (Figure 5b). Only in a situation like this can the phenomena of ICEK combine effectively with the native volumetric charge inside the dielectric end for engendering the most intensive concentration polarization layer to the best degree.

The above conjectures can be further witnessed by having a check at the specific simulation data of the output electric current in Figure 5a. From Figure 5a, the output current flux for the forward “off” working mode goes down as expected with the decrease of the negative interfacial charge, when considering a lower ion concentration in the IDZ from a reduced FZP (Figure 5c). Once the magnitude of surface charge density is smaller than σ_free-ideal_ = 0.001 C/m^2^, nevertheless, the electric current unexpectedly starts to increase as the surface charge further diminishes to zero (Figure 5a). So, although the backward current for the “on” working state decreases monotonously with a decaying interfacial charge due to fewer counterions in the native EDL at the dielectric end (the red line in Figure 5a), it is the nonmonotonic variation trend of the output current for the forward “off” working mode that determines the ultimate rectification behavior of the nanofluidic ion diode as a function of the negative interfacial charge on the dielectric walls. As a consequence, the rectification performance attains the best at an intermediate interfacial charge density of σ_free-ideal_ = −0.001 C/m^2^ (Figure 5b), and diminishes at either higher or lower surface charge densities. Most notably, the nanoscale ionic device even loses its rectification functionality once σ_free_ approaches −0.1 C/m^2^ or −0.0001 C/m^2^. Supported by above simulation results, there always exists an ideal interfacial charge density on the dielectric end to compete against the ICEK-induced bipolar IDL within the metallic end, for the metal-dielectric Janus ion-selective medium to work effectively as a nanofluidic ion diode. Numerical prediction provides an effective way to find this critical value of interfacial charge density for guiding a default set of parameters used in actual experiments.

**Figure 6:**
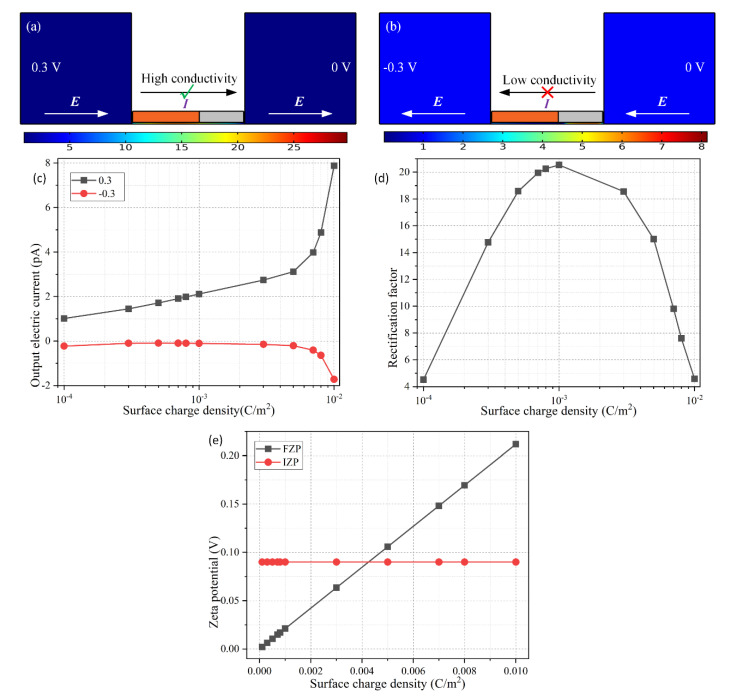
Effect of the native surface charge density of a *positively-charged* dielectric wall on the device rectification performance, under a given parametric space of *R_N_* =1 5 nm, *L_C_* = 600 nm, *V_S_* = ±0.3 V, and c_0_ = 0.1 mM. (**a**) A surface plot of the nondimensional electrolyte concentration for the “on” working state, in which the forward electric current diminishes due to the IEZ formed at bipolar junction at the central portion of the metallic nanopore. (**b**) A surface plot of the nondimensional electrolyte concentration for the “off” working state, in which the backward electric current is well forbidden due to the IDZ formed at another bipolar junction between the metallic and dielectric walls. (**c**) The forward and backward output electric current, and (**d**) the rectification factor, and (**e**) analytical estimation of the zeta potential magnitude, as a function of the surface charge density.

In addition to the influence of the magnitude of interfacial charge density, its polarity also exerts an important effect on the device rectification behavior (Figure 6). A careful inspection of Figure 6 tells us that a reversal of the surface charge polarity on the dielectric walls reverses the working state of the ion diode. That is, the forward “on” working mode in which the electric current is allowed to pass through the Janus nanopore (Figure 6a), and the backward “off” working state in which the current flux toward upstream is suppressed to great extent (Figure 6b), both of which are just the opposite case to the two working states of the Janus nanopore with a positive surface charge (Figure 4a,b). Moreover, the calculation data in Figure 6c–e are exactly the mirror image of Figure 5a–c, respectively. So, we believe that a reversal of surface charge polarity just inverts the direction of ion current rectification (Figure 3a,b compared to Figure 2b,c), while the rectification factor does not make a change in response to this (Figure 5b and Figure 6d). 

### 3.3. Effect of the Electrical Polarizability of the Conducting Wall

On the basis of the above analysis, nanofluidic devices with an opposite surface charge polarity of the dielectric wall can reflect the rectification functionality of one another by a simple operation of mirror symmetry. For such, we only need to focus on the rectification behavior of the Janus nanopore with a negatively-charged dielectric end, as shown in Figure 2b,c, so that the symmetric case with the dielectric wall of positive interfacial charges can be safely disregarded (Figure 3a,b). At the same time, an appropriate surface charge density of σ_free_ = σ_free-ideal_ = −0.001 C/m^2^ is chosen hereafter for producing a well-developed internal ICP interface at the bipolar junction driven by ICEK of the metallic end.

**Figure 7:**
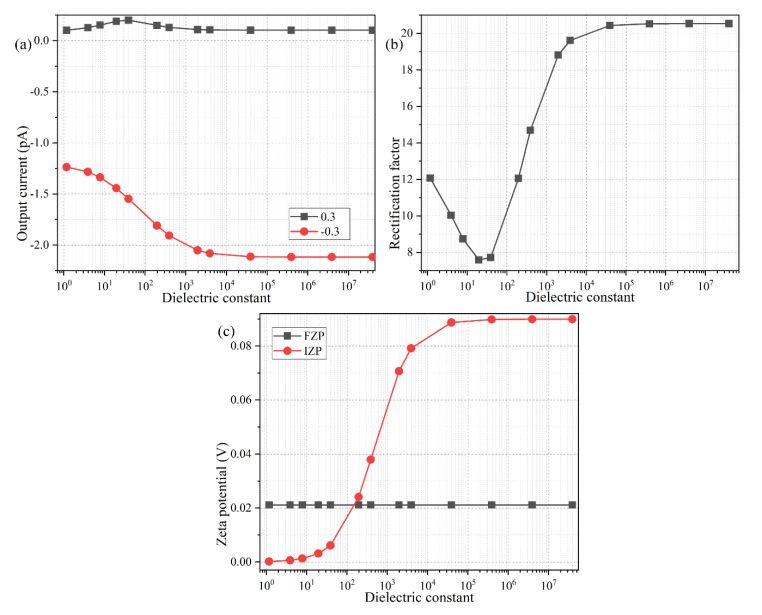
Effect of the relative dielectric constant of the conducting wall on the rectification behavior of the nanofluidic ion diode, under a given parametric space of *V_S_* = ±0.3 V, *L_C_* = 600 nm, σ_free_ = −0.001 C/m^2^, c_0_ = 0.1 mM, and *R_N_* = 15 nm. (**a**) The forward and backward output electric current, and (**b**) the resulted rectification factor, and (**c**) the analytical approximation of the FZP and IZP, as a function of the dielectric constant of the conducting wall of the metal-dielectric Janus nanopore.

From Equation (22), the electrical polarizability of the conducting wall exerts an important influence on the ICEK behavior within the Janus nanopore. Because the conducting layer is in serial connection with the double-layer capacitance skin on its surface, a higher dielectric constant of the conducting layer (approximated by a kind of solid material of varying permittivity but zero conductivity as in the conjugating condition of Equation (12)) would lower its electrical impedance and make less voltage drop within itself, therefore inducing a larger value of IZP (Figure 7c) and greater field-induced bipolar counterions along the conducting nanopore, which would bring enormous benefits for actuating a sufficiently strong ICP interface at the midchannel for ICEK-based ionic current control.

These qualitative physical pictures are demonstrated by quantitative simulation results in Figure 7a,b. Since a highly polarizable conducting material is required for effective field-effect ion current control, a peculiar variation behavior of rectification performance in the low permittivity range with ε_0_ ≤ ε_C_ ≤ 50ε_0_ is disregarded and not discussed in the present study (Figure 7a). By focusing our attention in the high permittivity range, it is discovered that an increase of the dielectric constant of the conducting wall will raise the output electric current in the backward “on” working mode, and restrain it in the forward “off” working state (Figure 7a), which is favorable for ionic current rectification using a metal-dielectric Janus nanopore (Figure 7b). The underlying reason is the enhancement of induced electrochemical polarization of the conducting wall with increasing dielectric permittivity, resulting in more bipolar counterions due to ICEK, so that ion enrichment/depletion at the field-induced bipolar junction within the nanochannel is evidently boosted with a highly polarizable conducting wall for the on/off working mode, respectively. As a result, it is preferable to develop a metal-dielectric Janus nanopore with the metallic end having the capability of being ideally polarized in an external DC forcing, such as a gold layer, in order to achieve the most ideal ICEK-enabled ionic current control, which agrees well with the conclusions from previous literature [73]. 

### 3.4. Influence of the Mismatch between Debye Length and Nanopore Size 

**Figure 8:**
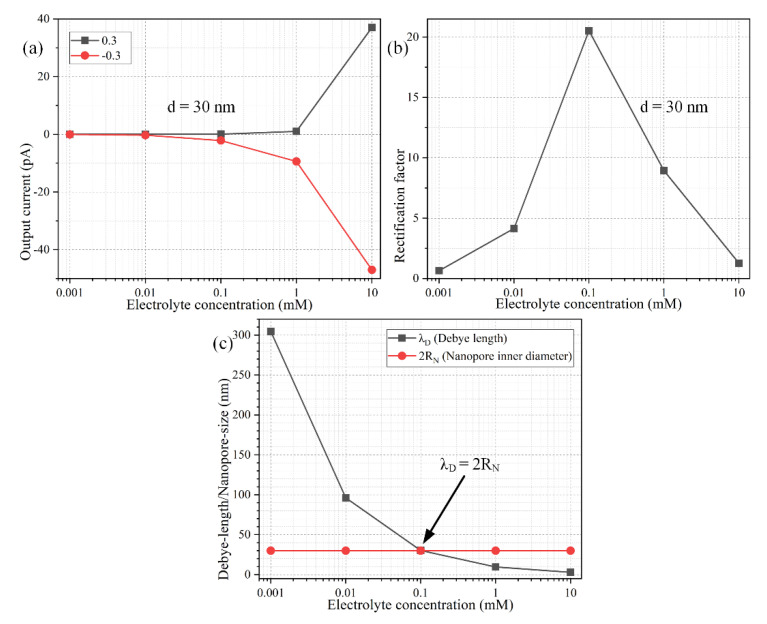
Influence of electrolyte concentration of the working fluid on the rectification performance of the nanofluidic ion diode, under a given parametric space of *V_S_* = ±0.3 V, *L_C_* = 600 nm, σ_free_ = −0.001 C/m^2^, and *R_N_* = 15 nm. (**a**) The forward and backward output ion current and (**b**) rectification factor, as a function of the background salt concentration. (**c**) Theoretical estimation of the electrolyte-concentration dependence of the Debye length, against the constant nanopore inner diameter.

From now on, it is assumed the Janus nanopore has always an ideally polarizable conducting end made of gold for maximizing the device rectification performance. We then test the effect that the concentration of background electrolyte has on the resulting diode functionality of the nanofluidic device (Figure 8). It is noteworthy that both the FZP (Equation (19)) and IZP (Equation (22)) are independent of the salt concentration in the buffer solution. Even so, the device performance in ionic current control driven by ICEK even becomes trivially small for ionic conductivity exceeding 0.153 S/m (c_0_ = 10 mM) due to the suppressed surface conductivity when the electrical double layer cannot well extend across the nanopore lateral dimension (Figure 8b).

Since the Ohmic current has a linear dependence on the local electric conductivity, the backward electric current for the “on” state increases with background salt concentration, albeit the ion enrichment zone in the middle of the conducting end is drastically inhibited with a shrink of the Debye length (the red line in Figure 8a). Meanwhile, the internal ion-depletion interface cannot be fully developed at the bipolar Janus junction between the metallic and dielectric end for the forward “off” working mode as well, resulting in a faster growth rate of output ionic current at a higher electrolyte concentration (the black line in Figure 8a). For such, as long as the thickness of the diffuse double layer does not match the inner diameter of the Janus nanopore (Figure 8c), the diode functionality will be greatly lowered, in that it is the uneven surface conduction of field-induced counterions that activates the internal interface of ICP within the confined space of bipolar Debye layer due to the action of ICEK, which can take place to the best extent only when the EDL extends completely inside the nanopore, namely, λ_D_ = 2*R_N_*. Any deviation of Debye length from the critical nanopore size (Figure 8c) will be detrimental to the device performance in ICEK-mediated ionic current control (Figure 8b). 

**Figure 9:**
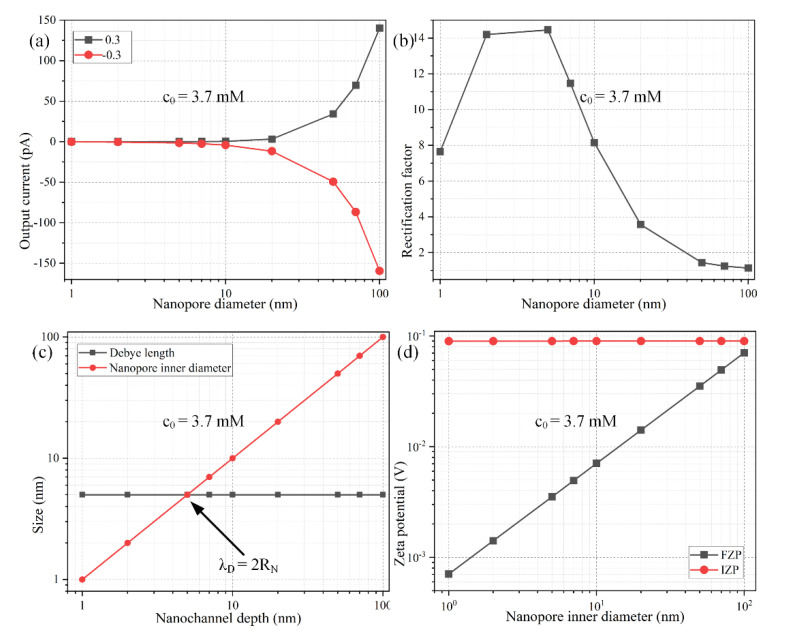
Effect of the nanopore inner diameter on the performance of ionic current rectification of the metal-dielectric ion-selective nanopore, under a given parametric space of *V_S_* = ±0.3 V, *L_C_* = 600 nm, σ_free_ = −0.001 C/m^2^, and c_0_ = 3.7 mM. (**a**) The forward and backward output ionic current and (**b**) the rectification performance as a function of the nanopore inner diameter. (**c**) A comparison of the Debye screening length with nanochannel depth for varying lateral dimension of the Janus nanopore. (**d**) Analytical approximation of FZP and IZP as a function of the nanopore inner diameter.

In this sense, because the double-layer thickness shrinks as the liquid conductivity increases (Figure 8c), for a given nanopore inner diameter, there is always a threshold salt concentration, only around which the nanoscale device can work as an effective nanofluidic ion diode with sufficient extension of the IDL full of bipolar counterions (Equation (26)). After simple transpositions, Equation (27) indicates the critical molar concentration of electrolytes is inversely proportional to the second power of the nanopore inner radius *R_N_*. Accordingly, as a buffer solution of higher ionic strength flows through the metal-dielectric ion-exchange medium, the lateral size of the cylindrical nanopore has to be made much smaller in the microfabrication process, in order for the nanopore’s inner diameter to approach the thinner Debye layer extension as much as possible, so that the ionic circuit platform can work with high efficiency for ICEK-based electric current control. That is, the application of a higher conductivity working fluid poses a higher requirement on the characteristic lateral dimension of the Janus ion-selective medium. For instance, for a background ion concentration c_0_ = 0.1 mM, the nanopore inner diameter should match 30 nm, which is just the Debye length for a liquid electrical conductivity of 0.00153 S/m, as used in the preceding simulation analysis. Once the background ion concentration is raised to c_0_ = 3.7 mM, however, the device rectification performance is maximized only when the nanopore inner diameter approaches the critical value of 5 nm (Figure 9a–c), which equals exactly the double-layer thickness for ion conductivity of 0.0565 S/m. Moreover, for a biological buffer with ion number density beyond 100 mM (1.528 S/m), the Debye screening length is lowered to 0.96 nm, so the nanopore inner diameter is going to have to be smaller than 1 nm to sufficiently sustain effective ICEK-mediated ion current control for practical on-chip biological applications. Above all, with a given electrolyte sample, the nanopore inner diameter should match the double-layer thickness as much as possible for realizing the most ideal diode functionality (Figure 8b,c and Figure 9b,c), albeit the value of fixed zeta potential (FZP) rises with an increase of the nanopore lateral dimension resulting in a weakened electrochemical polarization of the metallic end of the Janus nanopore (Figure 9d).

### 3.5. Influence of the Applied DC Voltage

We have employed a fixed source voltage of *V*_0_ = 0.3 V across a 1 μm long metal-dielectric Janus nanopore in previous sections. This is equivalent to an electric field strength of 0.3 V/μm, and resembles an AC voltage drop of 3 V across a 10 μm electrode gap adopted in typical experiments of AC electroosmosis [93]. In practice, the value of the DC voltage signal imposed on the source terminal may vary within a certain range according to the specific requirements of actual situation. So, it is then essential to clarify to what degree the magnitude of external DC bias applied across the S and D terminals affects the rectifying functionality of the nanoscale ion diode utilizing a metal-dielectric Janus nanopore.

**Figure 10:**
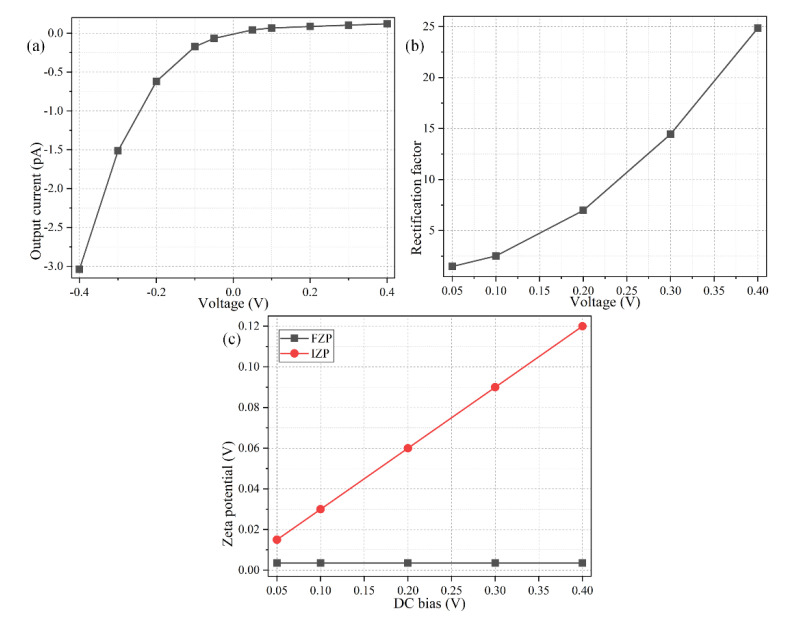
Effect of the imposed DC voltage across the S-D terminal on the rectifying functionality of the nanofluidic ion diode, under a given parametric space of *L_C_* = 600 nm, σ_free_ = −0.001 C/m^2^, c_0_ = 3.7 mM, and 2*R_N_* = λ_D_ = 5 nm. (**a**) Voltage-dependence of the output electric current. (**b**) Voltage-dependence of the rectification factor. (**c**) Analytical approximation of FZP and IZP as a function of the imposed DC bias across the nanopore length direction.

As shown in Figure 10a, with the increase of the positive DC bias from 0 to 0.4 V, the forward current for the “off” working mode increases according to the Ohmic law, but the growth rate becomes much smaller at a larger DC signal. On the other hand, the backward ionic current for the “on” operating mode increases much more quickly as the negative DC voltage signal becomes much larger from 0 V to −0.4 V (Figure 10a). The rectification performance of our nanoscale ion diode is calculated and presented in Figure 10b as a function of the source voltage. The *V*_0_-dependent rectification factor manifests as a nonlinear growth tendency. At a low DC voltage supply, the electrophoretic driving force exerted on the field-induced bipolar counterionic charges along the nanopore is weak for a small Dukhin number, leading to a nonideal rectifying behavior; on the contrary, with a further increase of *V*_0_, the device performance has a nonlinear growth trend, i.e., γ = V_0_^β^, with β > 1, since the slope of the curve becomes greater at a higher source voltage (Figure 10b).

This implies the electrochemical delivery of ICEK-tunable bipolar counterions via simultaneous electrophoretic and electroconvective mass transport serves as the main working principle of the presented nanofluidic ion diode (nonlinear electrokinetics), rather than the spontaneous unipolar Debye screening charge for compensating for the fixed interfacial charge at the dielectric wall (linear electrokinetics). Pertinent analysis sheds interesting lights on the importance of the phenomena of ICEK adjacent to the metallic wall during exposure to an external DC electric field in strengthening bipolar electrochemical polarization inside the Janus nanopore. An increase of the imposed DC signal is capable of inducing a stronger internal interface of depleted/enriched ICP for the “off”/“on” working state due to an enhanced IZP (Figure 10c), respectively, and thereby improves the resulting rectification performance of the proposed nanoscale ionic circuit platform as well. 

Although it has been demonstrated by numerical simulation that an increase in the applied DC voltage is in favor of improving the rectifying functionality, undesirable bipolar electrochemical reactions from phase transfer of electrons will take place on the surface of floating metallic wall for a relatively large IZP across the IDL at a higher *V*_0_ (Figure 10c). The effect of Faradaic current injection will bring vital damage to the metallic part of the Janus nanopore by causing galvanic erosions, which serves as the main limitation of the proposed method [94]. For above reasons, we should take great care with the choice of specific electrical signal for avoiding bipolar electrode reactions inside the metallic nanopore as much as possible, even though a higher imposed voltage to the source terminal may be theoretically profitable for getting a better rectification performance. 

## 4. Conclusions

In summary, by a delicate combination of bipolar ICEK phenomena around a floating metallic wall and spontaneous unipolar Debye screening adjacent to an insulating charged dielectric wall, we have proposed herein a unique way to generate reconfigurable surface-charge patterns in a metal-dielectric Janus nanopore, resulting in an on-chip nanofluidic ion diode for achieving effective ion current rectification by generating an internal ICP interface of opposite polarity for reverse working modes. According to the theoretical analyses and detailed discussions on the impact of a series of key parameters on the device rectifying behavior, the ability of ICEK-based ion current control is closely related to inhomogeneous surface electrokinetic transport of field-induced bipolar Debye screening charge within the IDL adjacent to the critical bipolar Janus interface between the metallic and dielectric wall. 

The most ideal rectification factor can be realized once the three following conditions are met simultaneously: firstly, the value of IZP across the IDL is no less than that of FZP across the native EDL; secondly, there is a sufficiently large area of the bipolar junction for the internal ICP interface to be fully developed; thirdly, the Debye screening length should match the nanopore inner diameter as much as possible for acquiring a moderate Dukhin number. More importantly, the geometric size of the presented nanofluidic device is highly scalable. For instance, the axial length of the asymmetric nanopore, thickness of metallic and dielectric wall, and size of microfluidic channels can get either larger or smaller according to our wishes, and the resulting diode functionality will not make an obvious change in response, as long as a sufficiently large DC voltage difference is imposed across the metal-dielectric Janus nanopore with an optimal length ratio of the metallic wall to the dielectric wall. This flexible scaling characteristic renders the nanoscale ion diode a qualified building block for miniaturized on-chip ionic circuit platforms. Supported by physical modeling, our theoretical demonstration makes it possible to better combine the ICEK mechanism with the confined space of nanochannels for developing novel nanofluidic devices in the context of small-scale liquid-phase integrate circuits and high-performance osmotic energy converters. 

## Supplementary Materials

The following are available online at https://www.mdpi.com/2072-666X/11/6/542/s1, Figure S1: Mesh-independence test of the numerical modeling of ICEK-based ion current rectification in a metal-dielectric Janus ion-selective medium, with a fixed parametric space of c_0_ = 0.1 mM, *Vs* = ±0.3 V, *Rn* = 15 nm, σfree = −0.001 C/m^2^, *L_C_* = 600 nm, *L_D_* = 400 nm. (a) Output electric current, and (b) rectification factor as a function of the maximum mesh size normalized with the Debye screening length within the nanopore.
