# Supplementary material for: A Simulation Analysis of Nanofluidic Ion Current Rectification Using a Metal-Dielectric Janus Nanopore Driven by Induced-Charge Electrokinetic Phenomena
Source: Micromachines (Basel). 2020 May 27;11(6):542. doi: 10.3390/mi11060542 (PMC7345169; doi:10.3390/mi11060542)
Supplement: Supplementary file 1 [file micromachines-11-00542-s001.pdf]

micromachines

Supplementary Materials: A Simulation Analysis of Nanofluidic Ion Current Rectification Using a Metal-Dielectric Janus Nanopore Driven by Induced-Charge Electrokinetic Phenomena

Weiyu Liu [1], Yongjun Sun [2,3,*], Hui Yan [2,*], Yukun Ren [2,3], Chunlei Song [2] and Qisheng Wu [1]

1 School of Electronics and Control Engineering, Chang'an University, Middle-Section of Nan'er Huan Road, Xi'an 710064, China; liuweiyu@chd.edu.cn (W.L.); qshwu@chd.edu.cn (Q.W.)
2 School of Mechatronics Engineering, Harbin Institute of Technology, West Da-zhi Street 92, Harbin 150001, China; rykhit@hit.edu.cn (Y.R.); sclei@hit.edu.cn (C.S.)
3 State Key Laboratory of Robotics and System, Harbin Institute of Technology, West Da-Zhi Street 92, Harbin 150001, China
* Correspondence: sunyongjun@hit.edu.cn (Y.S.); yanhui@hit.edu.cn (H.Y.)

Electronic Supplementary Information

This material gives supporting information of an investigation on the mesh-dependence trait of the simulation result by using the physical model introduced in the main text.

In order to demonstrate the effectiveness of our simulation model of ICEK-based ion current rectification in a metal-dielectric Janus nanopore, we have to test the mesh-dependence of the simulation results under different meshing scheme. As mentioned in the main text, the maximum mesh size h_{mesh} is asked to be no more than one-thirtieth of the Debye screening length within the metal-dielectric Janus nanopore. Extra fine triangular meshes of 0.05 nm in edge size are utilized at the entrance and exit of the nanochannel interfacing microchambers on both sides, for resolving the existence of an in-situ extended space charge layer (ESCL) wherein electroconvective ion transport plays an important role.

We change the maximum mesh size h_{mesh} step by step inside the nanopore, from half $\lambda_D/2$ down to one-fiftieth of the electrical double layer thickness $\lambda_D/50$, to confirm to what degree the mesh distribution exerts an influence on the simulation results. As shown in Figure S1a, the output electric current in the two opposite directions all converges to a corresponding stable plateau, so along as the maximum mesh size is no more than one-twentieth of the Debye length, i.e. $h_{mesh} \leq \lambda_D/20$. This variation trend also holds true for the simulation result of the rectification factor under a voltage contrast between −0.3 V and 0.3 V (Figure S1b), which changes by less than 3% as the grid size further decreases. As a consequence, it is reasonable to require the mesh size not to exceed one-thirtieth of the double-layer thickness, under the condition of which an ideal calculation accuracy can be guaranteed even with a confined computer resource.

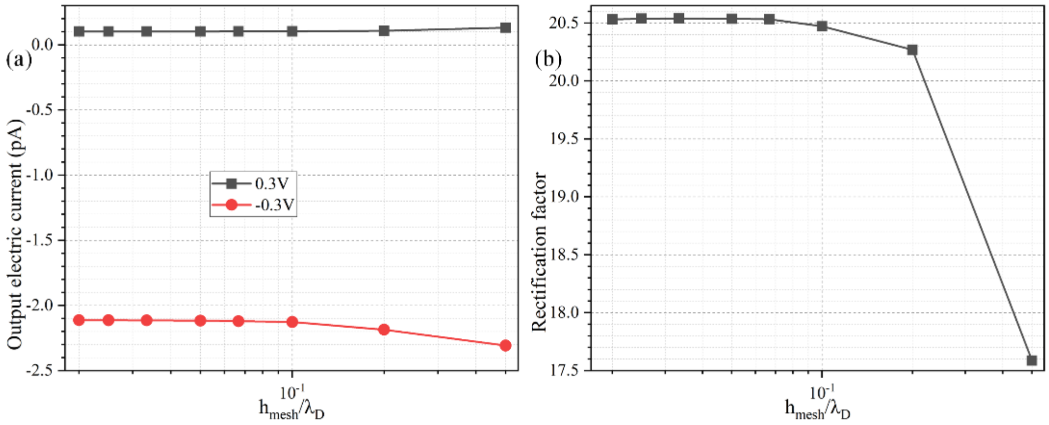

Figure S1. Mesh-independence test of the numerical modeling of ICEK-based ion current rectification in a metal-dielectric Janus ion-selective medium, with a fixed parametric space of c_0 = 0.1 mM, V_S = ±0.3 V, R_n = 15 nm, σ_{free} = −0.001 C/m^2, L_C = 600 nm, L_D = 400 nm. (**a**) Output electric current, and (**b**) rectification factor as a function of the maximum mesh size normalized with the Debye screening length within the nanopore.
